# Pediatric cardiac emergencies: Children are not small adults

**DOI:** 10.4103/0974-2700.76842

**Published:** 2011

**Authors:** Aisha Frazier, Elizabeth A Hunt, Kathryn Holmes

**Affiliations:** 1 Department of Pediatrics, Baltimore, Maryland, USA; 2 Division of Pediatric Cardiology, Baltimore, Maryland, USA; 3 Department of Anesthesiology and Critical Care Medicine and Division of Pediatric Anesthesiology and Critical Care Medicine, The Johns Hopkins University School of Medicine, Baltimore, Maryland, United States

**Keywords:** Aortic dissection, arrhythmias, cardiomyopathies, congenital heart disease, Kawasaki disease, myocarditis, pediatric cardiac emergencies, pulmonary embolism, sudden cardiac death

## Abstract

Compared with adults, cardiac emergencies are infrequent in children and clinical presentation is often quite variable. In adults, cardiac emergencies are most commonly related to complications of coronary artery disease; however, in pediatric cases, the coronaries are only rarely the underlying problem. Pediatric cardiac emergencies comprise a range of pathology including but not limited to undiagnosed congenital heart disease in the infant; complications of palliated congenital heart disease in children; arrhythmias related to underlying cardiac pathology in the teenager and acquired heart disease. The emergency room physician and pediatric intensivist will usually be the first and second lines of care for pediatric cardiac emergencies and thus it is imperative that they have knowledge of the diverse presentations of cardiac disease in order to increase the likelihood of delivering early appropriate therapy and referral. The objective of this review is to outline cardiac emergencies in the pediatric population and contrast the presentation with adults.

## INTRODUCTION

In adults, cardiac disease is not uncommon and the majority of causes of sudden cardiac death are due to atherosclerotic coronary artery disease.[1] However, cardiac death in children is a rare occurrence and the cause differs based on the age of the child. For infants less than one year of age, ductal dependent congenital heart disease is the most common cause, and after the first year and into early adulthood acquired heart conditions, such as myocarditis, cardiomyopathy and aortic dissection in patients with Marfan’s syndrome.[2] The objective of this review is to outline cardiac emergencies in the pediatric population and contrast the presentation with adults. The emergency room physician and pediatric intensivist will usually be the first and second lines of care for pediatric cardiac emergencies and thus it is imperative that they have knowledge of the diverse presentations of cardiac disease in order to increase the likelihood of delivering early appropriate therapy and referral.

## DUCTAL DEPENDENT LESIONS

Congenital heart diseases (CHD) are rare conditions, and the mortality is highest among infants and children and among the severe cardiac defects, such as hypoplastic left heart syndrome (HLHS). In the majority of the cases, mortality can be attributed to the underlying heart defect.[3] Infants with severe CHD can initially be asymptomatic, with a relatively benign physical exam prior to discharge from the newborn nursery, due to the patency of the ductus arteriosus. The ductus arteriosus usually closes within the first two weeks of life, but some take up to six. However, in infants with heart disease dependent on ductal patency, closure of the ductus can lead infants to become abruptly critically ill. It is important to realize then that any infant ≤ 6 weeks who presents in shock or profoundly cyanotic might be secondary to closure of the ductus arteriosus in conjunction with a right or left sided obstructive cardiac lesion.[4]

**Figure 1:**
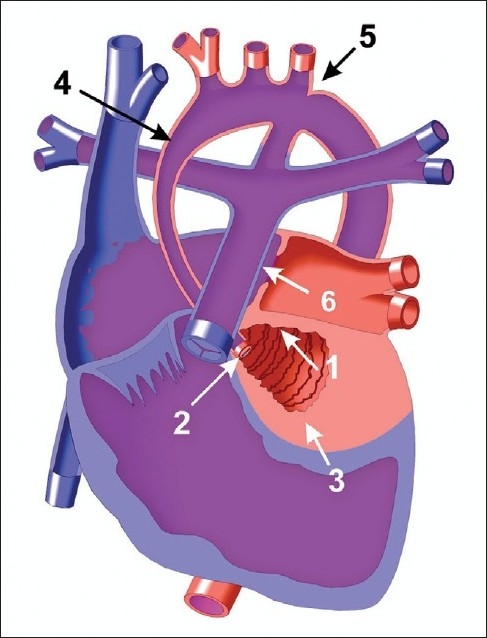
HLHS. 1. Atretic/stenotic mitral valve. 2. Atretic/stenotic aortic valve. 3. Hypoplastic left ventricle. 4. Hypoplastic ascending aorta. 5. Coarctation of the aorta. 6. Atrial septal defect. Systemic perfusion is provided by the PDA as pulmonary venous return to the left atrium shunts across the atrial septum, mixes with oxygen poor blood then flows to the pulmonary arteries to the lungs or systemic circulation via the PDA. Copied with permission from the illustrated field guide to congenital heart disease courtesy of scientific software solutions, inc

**Figure 2:**
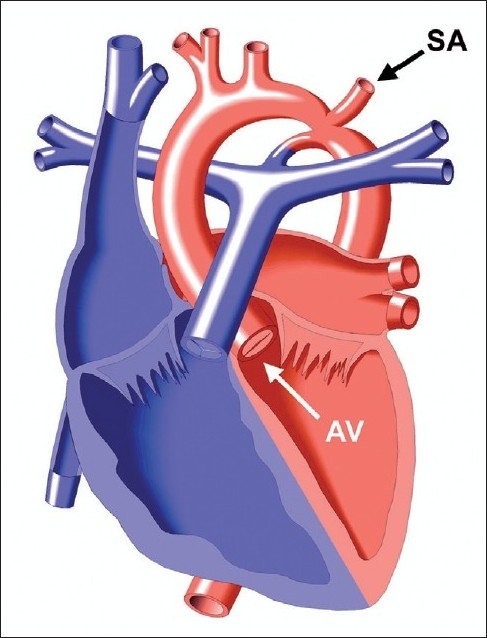
Coarctation of the aorta. Blood supply to the descending aorta is provided by ductal flow, thereby bypassing the aortic coarctation. Copied with permission from the illustrated field guide to congenital heart disease courtesy of scientific software solutions, inc

Left-sided obstructive lesions include HLHS, critical aortic stenosis, and coarctation of the aorta represent 12.4% of CHD.[3] In HLHS for example [Figure 1], as the PDA closes, pulmonary overcirculation develops and systemic hypoperfusion, circulatory collapse and shock ensue. The patient will present with respiratory distress, hepatomegaly, tachycardia, and signs of poor perfusion. Additionally, in critical coarctation of the aorta [Figure 2], femoral pulses are weak or absent and there is a blood pressure differential between the upper and lower extremities reflecting the gradient across the stenotic aortic segment. Prostaglandin infusion is necessary to reopen the PDA and basic resuscitative measures (ABC’s) should be initiated. Oxygen therapy can either help or hurt the patient and should be used judiciously, directed by the underlying anatomy and anticipated oxygen saturation for the heart defect. For example, to achieve a pulmonary circulation to systemic circulation flow ratio of 1:1, an oxygen saturation of 75 to 80% maybe desired (such as in HLHS). In this lesion, administration of oxygen to obtain a “normal” saturation of 100% would be detrimental, as the oxygen effect of reduced pulmonary vascular resistance compromises systemic perfusion.[5] Of note, in patients with critical coarctation, blood supply to the lower extremities will be extremely compromised such that an IO in the tibia may not be adequate and a right upper extremity or scalp IV may provide better access until a surgical cut down can be obtained.

Right-sided obstructive lesions such as severe tetralogy of Fallot and pulmonary atresia with intact ventricular septum depend on ductal patency to provide blood flow to the pulmonary artery. As the ductus closes, infants develop progressive and rapid cyanosis not improved with oxygen administration and respiratory distress. Prostaglandins should be started immediately to reopen the ductus arteriosus in order to provide pulmonary blood flow through left to right shunting (aorta across the PDA to pulmonary artery), as opposed to the right to left shunt on left-sided obstructive lesions. In these lesions, 100% oxygen is useful to improve pulmonary blood flow and increase dissolved O_2_ content.

## CONGESTIVE HEART FAILURE

Whereas ductal dependent lesions typically present early after ductal closure within the first two weeks of life, other CHD usually present in four-six weeks after birth when the pulmonary vascular resistance has fallen, such as a large ventricular septal defect.[6] Congestive heart failure (CHF) ensues from the significant left to right shunt and pulmonary congestion. Infants typically present with poor feeding, failure to thrive, tachypnea, and diaphoresis. Symptoms of older children are more typical adult symptoms of decreased exercise tolerance and dyspnea. Of note, dependent edema in children is very difficult to assess and more often a very late finding. Increased jugular venous pressure is difficult to assess in infants secondary to their short necks and liver size is used as a surrogate marker. CHF is usually related to one of the following conditions: anomalous left coronary artery arising from the pulmonary artery (ALCAPA), congenital cardiomyopathy, myocarditis, lesions resulting from a large intracardiac shunt (e.g. ventricular sepal defect) or extracardiac shunt (vein of Galen malformation), or arrhythmias.

**Figure 3:**
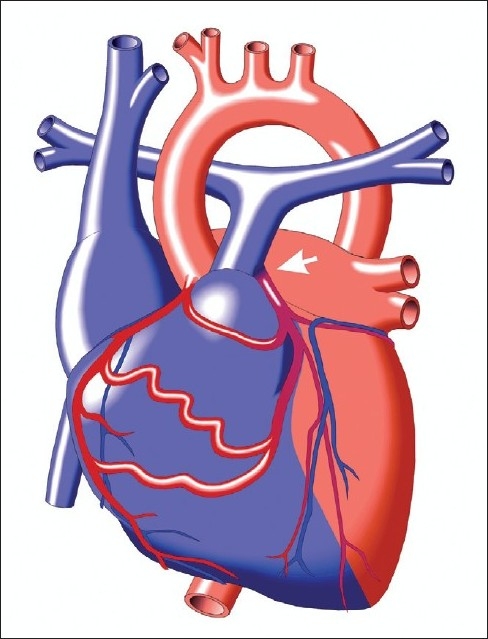
ALCAPA. The decreased PA pressure initially leads to a decrease in blood flow in the anomalous left coronary artery, then blood flow reverses (“coronary steal” phenomenon) leading to left ventricular insufficiency and infarction. Mitral regurgitation is a common finding from either annular dilation or infarction of the papillary muscle. Copied with permission from the illustrated field guide to congenital heart disease courtesy of scientific software solutions, inc

**Figure 4:**
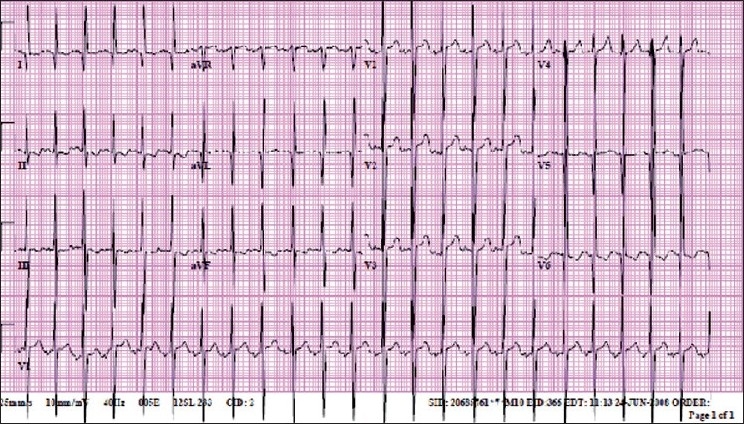
EKG of an eight-month-old who initially presented to the ER with respiratory distress and wheezing, and echocardiogram confirmed ALCAPA. EKG shows deep (≥3 mm) and wide (≥30 ms) Q waves in I, aVL, V6, absent Q waves in II, III, aVF, and ST segment changes

ALCAPA [Figure 3] is a type of CHD that typically presents at two to three months of age when pulmonary artery (PA) pressure decreases. ALCAPA is one of the most common causes of myocardial ischemia and infarction in children, specifically anterolateral infarct pattern[7] [Figure 4] and is associated with high mortality, where 90% of untreated patients die within the first year of life. Infants typically present with episodes of irritability (angina pain) and diaphoresis particularly during feeding (when myocardial oxygen consumption increases), signs of CHF, and a murmur of mitral regurgitation. Although mortality is high without treatment, prompt diagnosis and surgical treatment have excellent results with improvement in myocardial function overtime.

Some patients with ALCAPA may survive undetected through adolescence and adulthood due to the development of significant collateral circulation from the right coronary artery to the LCA. However, these collaterals may not be sufficient to perfuse the left ventricle, and these patients are at risk of chronic mitral regurgitation, ischemic cardiomyopathy, and sudden cardiac death from malignant ventricular arrhythmias in 80-90% of cases.[8,9] Surgical intervention is recommended, even in asymptomatic adult patients with ALCAPA and preserved left ventricular function discovered incidentally, due to the potential for progressive LV dysfunction and sudden death.[10]

## MYOCARDITIS

Myocarditis is an often insidious, inflammatory disease of the myocardium preceded by a non-specific flu-like illness. Myocarditis may have a fulminant presentation characterized by acute severe hemodynamic compromise with severe congestive heart failure or cardiogenic shock. It is the most common cause of dilated cardiomyopathy in children.[11] The symptoms can range from being asymptomatic to cardiovascular collapse and death. It is a potentially fatal disease, accounting for 22% of unexpected deaths in persons less than 20 years, with more staggering statistics for infants and children as high as 75 and 25% respectively. Recognition and diagnosis is imperative, but may prove challenging since the clinical presentation is varied and can be similar to other common conditions, such as gastroenteritis,[12] asthma and pneumonia. In one series, the initial evaluating physician diagnosed asthma or pneumonia in more than 50% of patients who actually had myocarditis.[13] In contrast, cardiac symptoms of chest pain are more common in adults.[14] In the European Study of Epidemiology and Treatment of Cardiac Inflammatory Diseases, 526 adults diagnosed with acute or chronic myocarditis of the 3,055 patients screened by endomyocardial biopsy, 32% presented with chest pain, 72% with dyspnea, and 18% had an arrhythmia.[15] In adults, myocarditis should be considered as a possible diagnosis among many cardiac syndromes, whereas in children the clinician needs to consider myocarditis among cardiac and non-cardiac presentations. ALCAPA, for example, should be on the differential in a two to three month old infant with presentations such as colic or respiratory distress, although EKG findings should help to differentiate them.[16] The long-term prognosis is favorable if recognized quickly and managed aggressively with a heart failure regimen and possibly mechanical circulatory support as a bridge to transplantation or recovery.[17–21]

## CARDIOMYOPATHIES

Cardiomyopathies are a heterogeneous group of myocardial diseases that causes significant disability and death. They are characterized by inappropriate ventricular hypertrophy or dilatation and are associated with electrical dysfunction or systolic and/or diastolic ventricular dysfunction.

## DILATED CARDIOMYOPATHY

Dilated cardiomyopathy (DCM) is a heart muscle disease characterized by dilation of the left ventricle and systolic dysfunction. Progressive heart failure ensues with resultant sudden and heart failure related death. DCM is the most common reason for heart transplantation in children and adults. In children, myocarditis is the most frequently identified cause followed by neuromuscular diseases,[22] whereas in adults the most common causes are idiopathic DCM, idiopathic myocarditis and coronary artery disease.[23,24] The long-term prognosis regardless of the cause may be progressive with approximately 54% of affected individuals within five years of diagnosis die or require a heart transplant.[23] The worst outcomes were found among older children, children who had CHF symptoms, and LV dysfunction at the time of presentation. Almost two-thirds of children present with congestive heart failure. Infants may present with failure to thrive, colic, or a conglomeration of symptoms of low cardiac output, such as poor perfusion, decreased distal pulses, pallor, and tachypnea, and on exam a diffuse or displaced cardiac apex, soft heart sounds, a gallop rhythm, hepatomegaly, and a mitral regurgitant murmur may be present. Adolescents may experience syncopal events, fatigue, exercise intolerance, dyspnea, pallor and tachycardia. GI symptoms of nausea, vomiting and abdominal pain are also frequently noted.[25,26]

## HYPERTROPHIC CARDIOMYOPATHY

**Figure 5:**
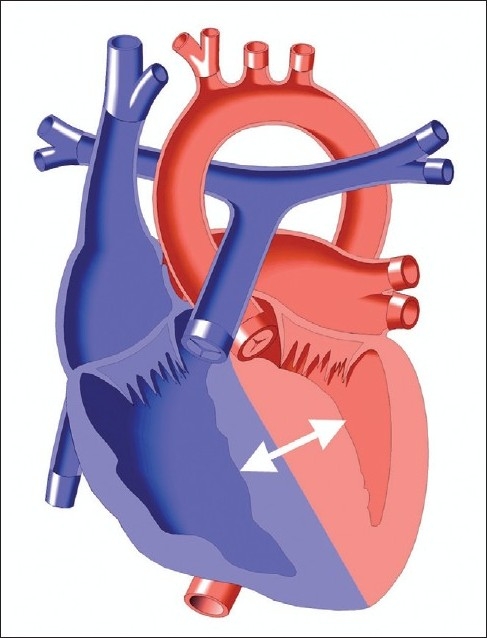
HCM is characterized by LV hypertrophy not due to another cardiac or systemic disease, hyperdynamic systolic function and impaired heart relaxation, with or without obstruction to blood flow across the left ventricular outflow tract. Copied with permission from the illustrated field guide to congenital heart disease courtesy of scientific software solutions, inc

Unfortunately cardiac arrest and sudden death may be the first presentation of Hypertrophic cardiomyopathy (HCM) [Figure 5]. It is the most common cause of sudden cardiac death in the young and can lead to progressive heart failure across all ages. The clinical presentation is varied. Young people, typically athletes aged 10-35 years old are the most common group to experience a cardiac arrest or sudden cardiac death. Evidence has shown that sudden death is primarily due to complex ventricular tachyarrhythmias,[27] bradyarrhythmias, and supraventricular arrhythmias that lead to ventricular fibrillation or hypotension, and these events are exacerbated when there is left ventricular outflow tract obstruction or ischemia.[28] Additionally, patients can present with symptoms related to poor diastolic function with preserved systolic function that include exertional chest pain and dyspnea, palpitations, presyncope, or syncope. The disease can then progress to end stage congestive heart failure heralded by systolic dysfunction, or patients can have atrial fibrillation associated complications, particularly embolic stroke.[29,30]

## ARRHYTHMIAS

Arrhythmias are far more common among adults than children, but are equally as important for the clinician be able to recognize the abnormal rhythm, respond immediately and treat appropriately. Of adults presenting to the emergency room with an acute myocardial infarction, 90% have a rhythm abnormality and 25% have a conduction disturbance.[31] Most pediatric arrhythmias occur among children with congenital and acquired heart disease, and symptoms of congestive heart failure or cyanosis precede the dysrhythmia. In children whose initial presentation is an arrhythmia, the underlying problem is a primary conduction disorder in a structurally normal heart. Children admitted to the cardiac intensive care unit for the medical management of heart disease, and particularly after cardiac surgery, certain catheterization procedures, and pre and post transplantation are at high risk for arrhythmias due to the underlying pathophysiology and medical and surgical interventions involved.[32]

The tachyarrhythmias can be characterized as supraventricular or ventricular. The most common symptomatic pediatric arrhythmia is supraventricular tachycardia (SVT) with an incidence of 1 in every 250 to 1,000 children. In infants, half are due to idiopathic causes, a quarter caused by congenital heart disease such as Ebstein’s anomaly, and 10-20% due to Wolff-Parkinson-White (WPW) syndrome, a type of AV reentrant tachycardia characterized on EKG by a delta wave, wide QRS, and short PR interval. It should be suspected if there is a regular and rapid heart rate usually greater than 180 bpm in older children and greater than 220 bpm in infants[33,34] On the electrocardiogram, the QRS is considered narrow complex with indiscernible p waves or retrograde p waves due to an accessory AV pathway. Patients with bundle branch block may be confused with VT. Most infants are hemodynamically stable in SVT, although within 48 hours congestive heart failure may develop with rapid deterioration occurring in 50%. The infant may appear lethargic, tachypneic, fussy, and mottled with signs of poor perfusion including cool extremities and hypotension. Older children rarely present in CHF and are usually able to articulate their symptoms of racing heart beat, chest pain, dizziness and lightheadedness. A 12 lead EKG and rhythm strip should be obtained and an immediate clinical assessment should be made to determine if the patient is cardiovascularly stable or unstable. For hemodynamically unstable SVT with poor perfusion and severe heart failure, adenosine (0.1 mg/kg/dose max 6 mg increased to 0.2 mg/kg/dose, max 12 mg), pushed and flushed quickly, can be given and/or immediate synchronized cardioversion (0.5 J/kg then increased up to 1 J/kg) if IV access is not immediately available. For stable SVT, vagal maneuvers such as valsalva or ice to the face may be attempted first. Adenosine can then be used if those maneuvers are unsuccessful.[35]

Ventricular fibrillation (VF) and pulseless ventricular tachycardia (VT) are the common causes of cardiac arrest in adults and often due to coronary artery disease. Fourteen percent of children compared to 23% of adults had VF or pulseless VT as the first documented rhythm during in-hospital cardiac arrest.[36] However, this is still much higher than many clinicians would have thought as the cause of cardiac arrest in children and demonstrates that those caring for children need to be capable of administering rapid defibrillation. The incidence of VF/VT as the first recorded rhythm in out of hospital arrest in children varies by age, from 4 to 5% in infants and children to 15% in adolescents.[37] In children, there is a cardiac origin in 21 to 71% of cases,[33] such as in palliated congenital heart disease and cardiomyopathies.[38] The incidence of VT after congenital heart surgery is low but can lead to a fatal outcome in an already hemodynamically unstable period. Intractable VT in the post operative period, particularly after surgery involving manipulation of the coronary arteries, (i.e. arterial switch operation for d-transposition of the great arteries) should raise suspicion for myocardial ischemia and the possible need for surgical revision.[39] In conduction disorders, such as in Long QT syndrome, torsades de pointes (a polymorphic VT) is characteristic and can degenerate into VF and sudden death.[37] WPW also can rarely lead to sudden death in high-risk patients after pre-excited atrial fibrillation with a rapid ventricular response that leads to VF.[40]

On EKG, VT is a wide QRS complex tachycardia that can be monomorphic or polymorphic, and VF has a fine or coarse chaotic rhythm with no discernible QRS complexes. Defibrillation is the treatment for VT/VF along with prompt and effective CPR.[41] There is clear evidence in adult cardiac arrest caused by ventricular arrhythmia that the rates of survival decreases with increasing time to defibrillation,[42,43] and mortality increases by 7-10% every minute defibrillation is delayed.[44]

## KAWASAKI DISEASE

Kawasaki disease does not usually present as a critical emergency; however, timely recognition and treatment can prevent the leading cause of acquired heart disease in children in the United States[45] and fatal cardiac events in adults. Kawasaki disease is an acute vasculitis of unknown cause that predominantly affects children under five years of age.[46] In 15 to 25% of untreated children, coronary artery aneurysms and progressive stenosis develop which can lead to significant morbidity and mortality from ischemic heart disease, myocardial infarction or sudden death.[47–49] The disease is diagnosed based on clinical criteria of at least five days of fever and at least four of the main clinical features including: polymorphous rash; limbic sparing non exudative conjunctivitis; mucocutaneous changes (e.g. strawberry tongue, dry cracked lips); cervical lymphadenopathy; and extremity changes with acute edema of the hands and feet or palmar erythema and after two to three weeks of illness periungual desquamation of the fingers and toes. There are also other supplemental laboratory and other clinical findings that are characteristic of the disease.

Children with a protracted febrile illness greater than five days with two to three of the clinical features of Kawasaki, incomplete Kawasaki should be considered. Moreover, the AHA recommends that an infant less than six months with fever greater than seven days and laboratory signs of systemic inflammation even without clinical features should have an echocardiogram.[50] Mortality is principally due to the cardiac sequelae. During the acute phase of the disease, in addition to coronary arteritis, pericarditis, myocarditis, and valvar heart disease can develop. Myocarditis can lead to myocardial dysfunction and children can present hypotensive and in cardiogenic shock. Arrhythmias can be associated as well as PR prolongation and nonspecific ST and T wave changes noted on electrocardiogram. There are specific guidelines associated with the acute and long-term management of Kawasaki disease.[50] Treatment consist of IVIG 2g/kg over 12 hours and high dose aspirin 80-100 mg/kg/day in 4 doses for 48 to 72 hours after fever defervescence, then low dose 3 to 5 mg/kg/day. Patients with persistent fever (≥ 36 hours after initial IVIG) or recurrent fever should be retreated with IVIG. The long-term management should be tailored based on the degree of coronary artery involvement and the risk for ischemic events.

## AORTIC DISEASE AND DISSECTION

Aortic dissection is rare in infants and children, and is associated with inherited disorders such as Marfan syndrome and other connective tissue disorders, and after balloon dilation of coarctation and re-coarctation, complication of cardiopulmonary bypass at the aortic cannulation site, and in young adults with a bicuspid aortic valve and ascending aortic dilation.[50,51] In contrast, atherosclerosis is the main cause of aortic aneurysms and dissection in adults, with hypertension being the main risk factor.

The clinical presentation in aortic dissection will vary based on the origin and extension of the dissection. Typically, there is abrupt onset of sharp pain that may change in location, followed by congestive cardiac failure due to severe aortic regurgitation. Patients may present with syncope, cardiac tamponade, stroke, limb ischemia with pulse deficits, paraplegia, and if renal or mesenteric arteries are involved may result in oliguria/anuria or have persistent abdominal pain. A diastolic murmur of aortic regurgitation and the typical wide pulse pressure is present in half of patients with proximal dissection. Aortic rupture into the pericardium with cardiac tamponade (i.e. friction rub, muffled heart sounds, hypotension, jugular venous distention, pulsus paradoxus) or the pleural space are indications for surgical emergencies.

A detailed history and physical exam should be performed; a patient with stigmata of Marfan syndrome or other connective tissue disease would raise the index of suspicion for dissection compared to other possible diagnoses (such as acute coronary syndrome, pericarditis, pulmonary embolism, and cholecystitis). An EKG is important to differentiate acute myocardial infarction to prevent the administration of thrombolytic therapy that would be detrimental in aortic dissection. Although, 20% of patients with dissection involving the ascending aorta have evidence of ischemia or infarction as the dissection extends into the coronary ostium, and in this situation, additional imaging studies should be preformed to confirm the dissection if the index of suspicion is high. Diverse imaging modalities can be used to detect aortic dissection. Transthoracic and transesophageal echocardiography can be useful in the emergency room and operating room for emergent decision making. CT scans are the most often used technique in patients with suspected aortic dissection and have a high sensitivity and specificity. Branch vessel involvement can be accurately identified with contrast angiography, and should be used in patients with neurological symptoms, acute renal failure, and limb and mesenteric ischemia.

Patients should be in an intensive care unit with blood pressure and heart rate monitoring. Fluid repletion, pain relief (e.g. morphine) and blood pressure lowering drugs, starting with a beta blocker and adding intravenous sodium nitroprusside for severe hypertension are important initial therapies. Surgical management in children is guided by similar principles as adults, with the emphasis on prophylactic intervention in patients with underlying dilation to prevent dissection.

## PULMONARY EMBOLISM

Pulmonary embolism (PE) is an uncommon cardiovascular emergency in children, although relatively common in adults. However, adolescent females with collagen vascular diseases or on birth control increase the risk as well as the increasing population of obese teenagers. Acute but often reversible right ventricular failure can result from pulmonary arterial occlusion by thromboemboli. The hemodynamic consequences of PE results when large and/or multiple emboli (occluding >30-50% of pulmonary arterial bed) acutely increase pulmonary vascular resistance, and the afterload that the right ventricular has to pump against is too high leading to RV failure. This can lead to sudden death or syncope with systemic hypotension that might progress to shock and death from acute RV collapse. The mortality rate is higher in children and they are more likely to be diagnosed at autopsy, where approximately 60% presented with sudden death. The most common predisposing factors were central venous catheters, malignancy, cardiac or other major surgery, and infection/sepsis.[52] Furthermore, thrombosis in children is more likely to originate in the upper extremity veins. Children typically present with dyspnea and hypoxemia, findings which are non specific and often difficult to differentiate from symptoms attributable to the underlying disease. Adolescents have similar presenting complaints to adults including pleuritic chest pain, dyspnea, cough and hemoptysis, although there is often a delay in the diagnosis in adolescents.[53]

The goal of diagnosis and appropriate treatment is to decrease mortality by preventing potentially lethal recurrent large thrombi. Clinical signs, symptoms and laboratory studies are not sensitive or specific to confirm or exclude PE, but should increase the index of suspicion to proceed with additional imaging. Such as, an electrocardiogram may show RV strain (classic S1,Q3,T3, new onset incomplete or complete RBBB), but this finding is not specific to PE and may be present in other causes of RV strain.

Diagnostic imaging include venous ultrasonography to detect DVT; echocardiography to detect right heart thrombi, a right to left shunt through a patent foramen ovale (an indication of elevated right sided pressures), and right ventricular dysfunction (RV dilation, hypokinesis, increased velocity of the tricuspid regurgitation jet) findings which were associated with greater than two-fold increased risk of PE-related mortality;[54] V/Q lung scan is useful when the scan is normal or indicates high probability, but most patients do not have findings that are definitive;[55] Pulmonary angiography is the gold standard, although it is an invasive specialized technique with associated risks that should be reserved for the small subgroup of patients in whom the diagnosis of PE cannot be made by other non invasive imaging modalities.

Heparin followed by oral anticoagulation therapy is the standard treatment for PE. In children, the use of thrombolytic therapy, such as tissue plasminogen activator (tPA), and surgical or transvenous catheter embolectomy may be considered in hemodynamically unstable patients at high risk of death due to RV failure. This can quickly reduce the RV afterload by recanalization of the occluded pulmonary arterial bed.[56]

## CONCLUSION

While there are some similarities in cardiac disease in children and adults, there are some very important differences as well. Clinicians who care for children, even occasionally, should be aware of diseases that are unique to children (i.e. ductal dependent lesions) as well as the clinical presentations that are more unique to children (i.e. colic, sweating with feeds, hepatomegaly, differences in 4 extremity blood pressures, etc…). In addition, they should be aware of some of the approaches to therapy that differ from that of adults with cardiac disease (i.e. prompt administration of prostaglandins in ductal dependent lesions and the need to administer oxygen judiciously in children whose CHD leads to usual saturations of 75 to 85%). Prompt recognition and treatment of cardiac emergencies and consultation of pediatric intensivists and cardiologists in children can lead to improved outcomes.
